# Intense and unpredictable perturbations during gait training improve dynamic balance abilities in chronic hemiparetic individuals: a randomized controlled pilot trial

**DOI:** 10.1186/s12984-020-00707-0

**Published:** 2020-06-17

**Authors:** Vahid Esmaeili, Andréanne Juneau, Joseph-Omer Dyer, Anouk Lamontagne, Dahlia Kairy, Laurent Bouyer, Cyril Duclos

**Affiliations:** 1 School of Rehabilitation, Université de Montréal, P.O. Box 6128, Station Centre-Ville, Montreal, Quebec H3C 3J7 Canada; 2 Centre for Interdisciplinary Research in Rehabilitation-Institut Universitaire sur la Réadaptation en Déficience Physique de Montréal, in CIUSSS du Centre-Sud-de-l’ile-de-Montréal, Montreal, Canada; 3 Lethbridge-Layton-MacKay Rehabilitation Centre, Montréal, Canada; 4 School of Physical and Occupationnal Therapy, McGill University, Montréal, Canada; 5 Department of Rehabilitation, Faculty of Medicine, Université Laval and Center for Interdisciplinary Research in Rehabilitation and Social Integration, CIUSSS-CN, Quebec City, Canada

**Keywords:** Stroke, Perturbation training, Balance, Gait, Strength, Community mobility

## Abstract

**Background:**

Previous studies have assessed the effects of perturbation training on balance after stroke. However, the perturbations were either applied while standing or were small in amplitude during gait, which is not representative of the most common fall conditions. The perturbations were also combined with other challenges such as progressive increases in treadmill speed.

**Objective:**

To determine the benefit of treadmill training with intense and unpredictable perturbations compared to treadmill walking-only training for dynamic balance and gait post-stroke.

**Methods:**

Twenty-one individuals post-stroke with reduced dynamic balance abilities, with or without a history of fall and ability to walk on a treadmill without external support or a walking aid for at least 1 min were allocated to either an unpredictable gait perturbation (Perturb) group or a walking-only (NonPerturb) group through covariate adaptive randomization. Nine training sessions were conducted over 3 weeks. NonPerturb participants only walked on the treadmill but were offered perturbation training after the control intervention. Pre- and post-training evaluations included balance and gait abilities, maximal knee strength, balance confidence and community integration. Six-week phone follow-ups were conducted for balance confidence and community integration. Satisfaction with perturbation training was also assessed.

**Results:**

With no baseline differences between groups (*p* > 0.075), perturbation training yielded large improvements in most variables in the Perturb (*p* < 0.05, Effect Size: ES > .46) group (*n* = 10) and the NonPerturb (*p* ≤ .089, ES > .45) group (*n* = 7 post-crossing), except for maximal strength (*p* > .23) in the NonPerturb group. Walking-only training in the NonPerturb group (*n* = 8, pre-crossing) mostly had no effect (*p* > .292, ES < .26), except on balance confidence (*p* = .063, ES = .46). The effects of the gait training were still present on balance confidence and community integration at follow-up. Satisfaction with the training program was high.

**Conclusion:**

Intense and unpredictable gait perturbations have the potential to be an efficient component of training to improve balance abilities and community integration in individuals with chronic stroke. Retrospective registration: ClinicalTrials.gov. March 18th, 2020. Identifier: NCT04314830.

## Background

Post-stroke impairments, particularly those affecting dynamic balance, are responsible for a fall incidence rate as high as 37 to 73% during the first year after stroke [1–3]. Dynamic balance can be defined as the ability to achieve, maintain, or restore the line of gravity within the continuously changing base of support [4, 5]. Dynamic balance impairments in individuals post-stroke are due to decreased sensory information and muscular strength on the paretic side, [5] slow gait speed, [6] reduced adaptability to constraints, [7] impaired timing of muscle activation [2] and delayed or disrupted postural responses [8, 9]. Impaired dynamic balance and related falls result in psychological and physical consequences such as reduced socialization and activity, fear of falling and fractures [10].

Effective dynamic balance training post-stroke should include balance perturbations during gait [11, 12]. Non-specific training approaches with mobility exercises improve functional balance and mobility in persons with stroke, [13, 14] but the effects are small [15]. In addition, individuals post-stroke predominantly fall during gait [10] where compensatory strategies that are essential for balance recovery require activation of neural pathways specific to involuntary postural responses [16]. On the other hand, the stepping strategy, i.e. taking a step, or changing its characteristics, to maintain balance, is essential for counteracting unpredictable situations leading to falls while walking in ordinary life [16]. To trigger this strategy, perturbations should be unpredictable and intense enough to be challenging [17].

While gait perturbation training has already been reported as an effective method for reducing fall rates in older adults, [17, 18] there is limited evidence on the effectiveness of perturbation training in individuals post-stroke [19–21]. Two recent studies used perturbations in a standing position, which had a limited effect on balance abilities, similar to control, traditional balance training [19, 22]. Another study that used low-amplitude perturbations did not trigger large stepping responses [20]. Lastly, gradual increases in treadmill speed of walking during the training sessions may be a confounder in these studies, [20, 21] given that treadmill gait training is known to improve gait abilities [23] and possibly balance [24, 25]. To determine whether gait perturbations are effective in clinically improving dynamic balance, it is necessary to control for the effect of gait training on gait and balance abilities. In addition, perturbations that occur in daily life vary in intensity and require specific adaptations in stepping reactions or gait pattern. It therefore seems necessary to include medium-to-large perturbations in training programs to challenge gait adaptability in individuals with stroke.

The purpose of this pilot study was therefore to compare the effects of gait training with and without unpredictable perturbations that trigger stepping reactions on dynamic balance and gait abilities in individuals with chronic stroke. We also measured possible sustained improvements in balance confidence and reintegration into the community 6 weeks after the end of each program. We hypothesized that the experimental perturbation training (Perturb) group would improve in dynamic balance, walking speed, balance confidence and muscle strength. These effects would facilitate the transfer of improved balance abilities towards better community integration [20]. The control group (NonPerturb), which would walk on the treadmill without perturbation, would only improve in walking speed, and possibly dynamic balance, but to a lower extent due to the lower-level challenge of the steady treadmill speed throughout the training program [21, 25]. The participants in this group who would cross over to perturbation training once the no-perturbation training was finished, would demonstrate greater improvements in balance and gait abilities during this second training period.

## Subjects and methods

A convenience sample of 21 individuals with a chronic unilateral stroke (> 6 months) was recruited and allocated to two groups (2 females in each group): Perturb and NonPerturb. In the absence of preliminary data, sample size/power were not calculated a priori. Inclusion criteria included reduced dynamic balance abilities (MiniBESTest score below the lower limit of the 95% confidence interval of normative data according to age group [26]) with or without a history of falls and the ability to walk on a treadmill without external support such as handrails or a walking aid for at least 1 min. Exclusion criteria included hemineglect (more than 6 omissions on the Bells cancellation test) [27], major cognitive impairment (Mini-Mental State Examination score below 24/30), [28] uncorrected visual deficit or pathologies other than stroke affecting gait or balance. Clinical characteristics, such as socio-demographic data (age, sex, time since stroke) were obtained from the participants’ medical charts and interviews. The Chedoke McMaster Stroke Assessment (CMSA) was used to determine motor impairments at the foot and leg [29]. Spasticity was evaluated using the composite spasticity index at the hip, knee and ankle [30]. The Consolidated Standards of Reporting Trials (CONSORT [31]) and Template for Intervention Description and Replication (TIDieR [32]) checklists were used to prepare this manuscript.

**Fig. 1:**
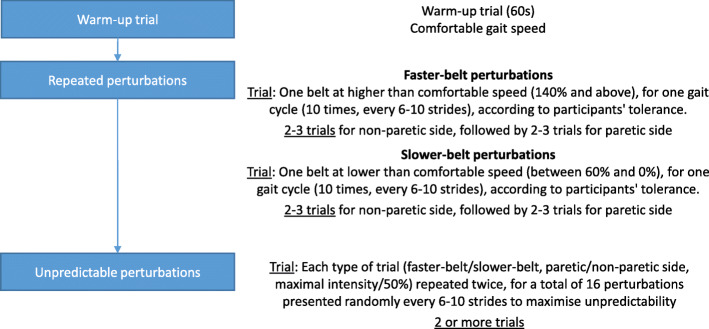
Description of the content of one training session

The participants attended nine training sessions (Fig. 1) over 3 weeks. A split-belt treadmill (Bertec Fit®) was used to induce perturbations one gait cycle at a time by changing the speed of each belt independently. Each perturbation training session began with a 60-s walking period at a comfortable treadmill speed. First, the same type of perturbation was applied repeatedly (i.e., same type of perturbation, repeated with the same intensity, but unpredictable in time). Then, unpredictable perturbations were applied (i.e., type, intensity and time of the perturbation was unpredictable). When the perturbations were repeated, 10 perturbations were applied during one trial at the same intensity level, set as a percentage of the comfortable gait speed. By increasing or decreasing the speed of one of the belts by a percentage of the comfortable gait speed (140,160,180%... or 60, 40, 20 and 0%), different intensities and types of perturbations could be produced (i.e., faster-belt or slower-belt perturbations). Faster-belt perturbations simulated trips and slower-belt perturbations simulated slips [33]. The maximal intensity of the perturbations was chosen when the gait pattern became altered due to large stepping reactions and/or the participant’s tolerance, i.e. whether he/she accepted or not to increase the intensity of perturbation. Each participant had three faster-belt, repeated perturbation trials followed by three slower-belt, repeated perturbation trials that increased in difficulty. These trials were first conducted on the non-paretic side and then on the paretic side, with perturbations being applied every 6 to 10 strides. Unpredictable perturbation trials included perturbations on either side, at the highest intensity level and 50% of the highest intensity of faster-belt and slower-belt perturbations reached during the repeated perturbation trials. Each of these perturbations were repeated twice, for a total of 16 perturbations per unpredictable perturbation trial (two sides, two levels of difficulty for fast- and slower-belt perturbations, each repeated twice). The number of unpredictable perturbation trials depended on each participant’s tolerance, i.e. he/she agreed to have another trial. The intensity of the perturbations also gradually increased with each session based on the participants’ tolerance. A harness was used to prevent a fall during training without providing any body weight support during gait or the perturbations.

Participants in the NonPerturb group only walked on the treadmill at their comfortable treadmill speed. The duration of the training sessions (walking time) for each participant in the NonPerturb group was matched against that of a participant in the Perturb group with a similar over ground speed. NonPerturb participants were offered a chance to cross over to the experimental treatment at the end of the control intervention. To limit the effect of the gait training itself, treadmill speed between perturbations was not increased across the nine sessions of each training program.

The following two primary outcome measures were used to evaluate the effects of the training programs: 1) The Mini-BESTest was used to assess balance abilities in dynamic conditions while performing 14 dynamic tasks, categorized in four subsystems (anticipatory activity, reactive postural control, sensory orientation and dynamic gait) [34]. The Minimal Clinically Important Difference (MCID) of the Mini-BESTest in a chronic stroke population is 4/28 points [35]. 2) The 10-Meter Walk Test was used to evaluate gait speed at comfortable and fast over ground speeds, which, on its own, is a good indicator of level of independence [36]. The MCID of the 10-Meter Walk Test is 0.14 m/s in the stroke population [37].

**Fig. 2:**
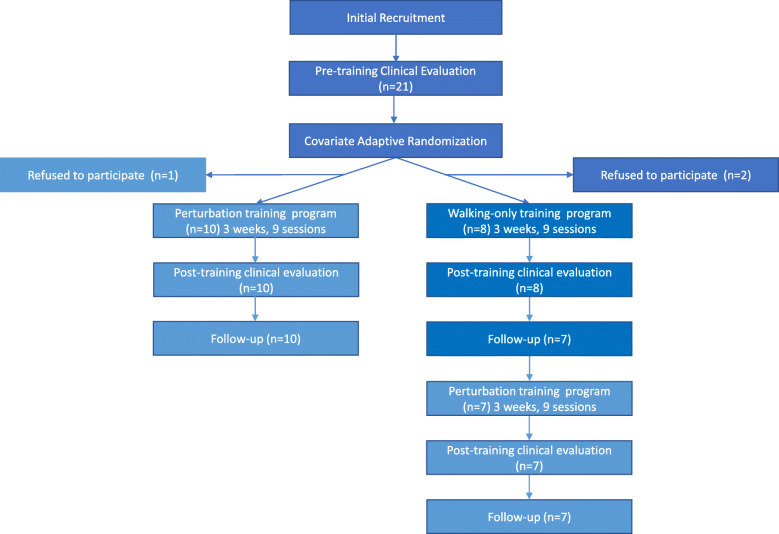
Flow diagram of the study; text boxes with a light blue background highlight the perturbation training periods

In order to better understand how the training programs could potentially improve balance and gait abilities, we also evaluated the following secondary outcome measures. Maximal muscle strength was evaluated at the paretic and non-paretic knee extensors using a Biodex dynamometer in isometric conditions at 90° knee flexion. Balance confidence was evaluated using the Activity-specific Balance Confidence (ABC) scale. This questionnaire assesses how confident individuals are in maintaining balance during 16 tasks on a 0–100% scale, with 100% being completely confident [38]. The Reintegration to Normal Living Index (RNLI) is a questionnaire that was used to determine whether the training programs had an effect on the daily lives of the participants. A lower score represents better integration. The MCID of the RNLI is 7% [39]. Both the ABC and RNLI tools have appropriate psychometric properties in a chronic stroke population [38, 40]. Clinical and strength assessments were performed in the week before and the week after the end of the training programs by different evaluators trained in the use of these evaluation tools and blinded to group assignment and time of assessment. Balance confidence and reintegration into social activities were re-evaluated 6 weeks after the end of the training program via a phone interview (Fig. 2) to evaluate the sustained effect of the intervention. In addition, the level of satisfaction with the perturbation training program was evaluated using the Short Feedback Questionnaire (SFQ) [41] modified for perturbations (SFQ-Mp) in the week following the end of the perturbation training. The questionnaire items with a five-point rating scale are presented in Fig. 5. NonPerturb participants who crossed over to the non-perturbation training program after the 6-week follow-up phone interview were also evaluated clinically before, immediately after and 6 weeks after the second training program as were the participants in the experimental Perturb group (Fig. 2). Data collection and training sessions were performed at the Gingras-Lindsay rehabilitation institute in Montreal, Canada. The study received ethical approval from the Research Ethics Committee of the Centre for Interdisciplinary Research in Rehabilitation of Greater Montreal. All participants signed a consent form prior to study enrollment.

To reduce the risk of potential bias due to small sized groups, we used a covariate adaptive randomization process, [42] with the following baseline characteristics tentatively matched between groups: dynamic balance abilities, comfortable and fast over ground gait speed, age, motor impairments of the leg and foot, height, and weight. An initial Perturb subgroup was recruited for perturbation training. Then new participants were placed in either the NonPerturb or the Perturb group depending on matching characteristics. Blinding of the participants or the person allocating the individuals to groups was not possible due to the nature of the intervention and the design of the study (partial cross-over). The two experimenters, who were trained physical therapists and were supervising the training sessions were also not blinded regarding the intervention.

Baseline and post-training data were compared within the Perturb (perturbation) and NonPerturb (walking-only and perturbations post-cross over) groups to show the effects of each training using Wilcoxon tests. In addition, balance confidence and RNLI scores measured at the 6-week follow-up were compared to pre-training and immediate post-training values using Wilcoxon tests to estimate immediate and six-week effects of each training program. Associated effect sizes (r) were calculated using the Z value of the Wilcoxon signed-rank test (r = Z/√N) [43]. We compared primary and secondary outcomes at baseline, immediately following the training program (and at the 6-week follow-up for ABC scale and RNLI only) using Mann-Whitney U tests to show 1) whether the clinical characteristics of the groups differed before training, and 2) whether the perturbation programs (Perturb and NonPerturb groups) resulted in better performance post-training and at follow-up than the walking-only program (NonPerturb group only). In addition, we compared the scores obtained for each subsystem of the Mini-BESTest at baseline and immediately after the training programs, using Wilcoxon tests, to determine whether subsystems of balance were specifically improved by the perturbation or walking-only training programs.

## Results

No significant differences were found before training between the Perturb group and the NonPerturb group (Mann-Whitney U test; walking-only: *p* > 0.075 (Table 1), and secondary perturbation training program after cross over: *p* ≥ .135).

**Table 1:** General characteristics and clinical scores at baseline for the Perturb and NonPerturb groups

Baseline characteristics	Perturb group (median (IQR))	NonPerturb group (median (IQR))	*p* value
Height (in cm)	173.0 (20.0)	170.5 (16.0)	.656
Weight (in kg)	83.2 (25.0)	77.9 (9.4)	.477
Age (in years)	58.0 (6.7)	57.5 (18.0)	.964
Months post stroke	67.5 (19.0)	104.5 (137.0)	.075
Chedoke leg (/7)	5.0 (1.5)	5.0 (2.75)	.829
Chedoke foot (/7)	3.0 (2.0)	2.0 (5.0)	.573
Hip spasticity	2.0 (0)	2.0 (2.5)	.882
Knee spasticity	4.5 (2.8)	4.5 (5.3)	.964
Ankle spasticity	4.0 (1.5)	5.0 (3.3)	.360
Dynamic balance (/28)	20.0 (2.75)	16.5 (6.25)	.447
Comfortable over ground speed (in m/s)	0.90 (0.31)	0.96 (0.50)	.689
Fast over ground speed (in m/s)	1.35 (0.57)	1.24 (0.46)	1.000
Paretic knee extensors, maximal strength (in Nm)	94.5 (46.6)	113.9 (45.9)	.165
Non-paretic knee extensors, maximal strength (in Nm)	139.1 (51.4)	123.2 (49.2)	.643
Balance confidence (/100)	75.9 (31.3)	65.9 (17.5)	.398
Reintegration to normal living index (/22)	3.0 (4.5)	2.0 (1.75)	.591

*Perturb* Perturbation group, *NonPerturb* non-perturbation group (Walking-only), *IQR* Interquartile range

All participants attended the nine sessions in each of their training programs. The average duration of participation in the training periods were 21.6 (8.2) (Mean (SD), Perturb), 18.3 (5.0) (NonPerturb walking-only), 20.1 (3.3) (NonPerturb, perturbations) days. The follow-up periods were 60.1 (23.2) (Perturb), 55.5 (12.9) (NonPerturb walking-only), 91.4 (38.9) (NonPerturb, perturbations) days. The duration between the follow-up of the first training period and first day of the second period training for the NonPerturb group was 45.4 (27.4) days. Various delays due to medical or personal reasons increased training duration or time between training in 4 participants. One participant allocated to the Perturb group and two participants in the NonPerturb group refused to participate in the project after allocation (Fig. 2). Given that the recruitment and training stopped in the winter due to inclement weather, the perturbation training post-cross over for the NonPerturb group was often delayed and done mostly during the following summer.

**Fig. 3:**
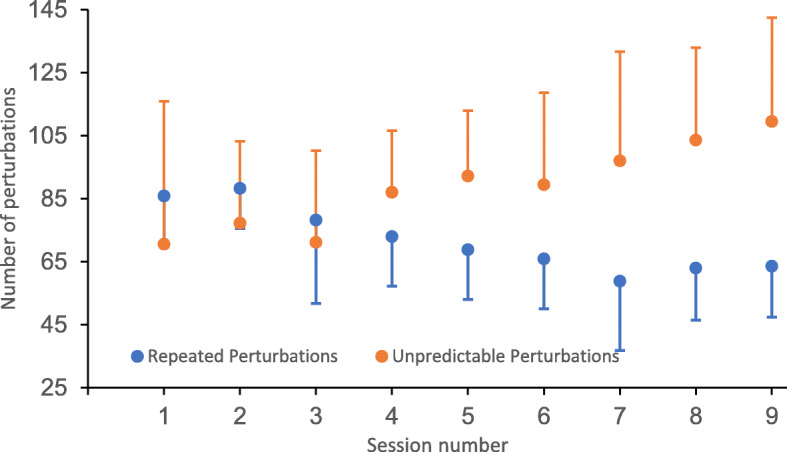
Mean and standard deviations (error bars) in the number of repeated (blue) and unpredictable (orange) perturbations applied among the 17 participants (Perturb group: *n* = 10, NonPerturb group after crossover: *n* = 7) who received perturbation training

On average, the total number of repeated and unpredictable perturbations applied over the nine sessions scheduled in the training program for each subject reached 618 (183) and 768 (237), respectively, with a progressive increase in the number of unpredictable perturbations and a decrease in the repeated perturbations throughout the training program (Fig. 3). The highest intensity of faster-belt and slower-belt perturbations was 280 and 0% respectively, except for three participants who did not reach such a level of difficulty. Participants reached the first slowest-belt perturbation (0%) between the 2nd and the 6th training session (mean (SD): 3.8 (1.6)). The corresponding values for the fastest-belt perturbation (280%) were observed between the 4th and 9th training sessions (mean (SD): 6.7 (1.4)). Neither perturbation training nor walking-only training worsened spasticity (Wilcoxon *p* ≥ .257 for composite spasticity index scores at the hip, knee and ankle). The duration of each session ranged from 35 to 70 min depending on gait cadence and the amount of rest the participants required.

### Effects of perturbation training vs. walking-only training

**Fig. 4:**
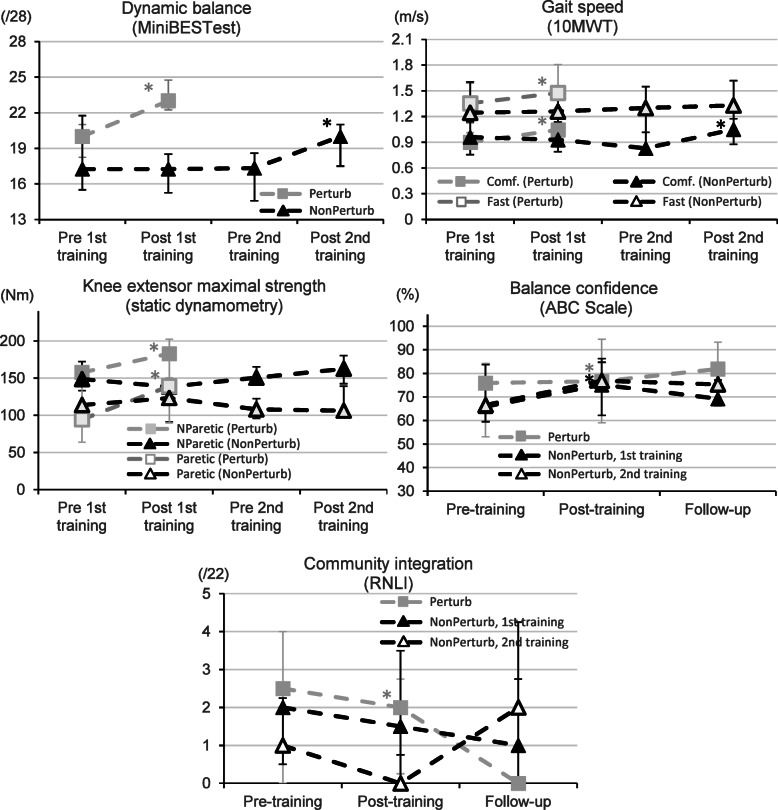
Effects of perturbation (Perturb training (grey) and NonPerturb 2nd training (solid black)) and walking-only training (NonPerturb 1st training (black outline)) on dynamic balance (Mini BESTest, top left), walking speed (10 MWT, top right), maximal knee extension strength (dynamometry, middle left), balance confidence (ABC, middle right) and level of community reintegration (RNLI, bottom) pre-, and immediate post-training, as well at the 6-weeks follow-up for balance confidence and community reintegration. NParetic: Nonparetic side. * indicates statistically significant change compared to the previous assessment time

Perturbation training led to large improvements in dynamic balance and comfortable and fast over-ground speeds in the Perturb group (Table 2, Fig. 4). This increase was equal or above the MCID level for dynamic balance in 5/10 participants (+ 6 (2)/28) vs. only 1/8 in the walking-only group, and equal or above the MCID level for comfortable speed in 6/10 participants, vs. 0/8 in the walking-only group. Concerning the subsystems of balance control, only anticipatory activity score increased significantly with both training programs (Perturbation (*n* = 17) (Median (interquartile range)): 3.0 (2.0) to 4.5 (2.5)/6 (*p* = 0.006) vs. Walking-only: (*n* = 8): 3.0 (2.0) to 4.5 (2.3)/6 (*p* = 0.011)). Reactive postural control (3.0 (2.5) to 3.9 (2.5) (*p* = 0.039) vs 2.4 (4.0) to 1.5 (4.5) (*p* = 1)), and dynamic gait scores (6.0 (3.0) to 8.0 (2.5)/10 (*p* = 0.002) vs 7.0 (1.8) to 6.5 (2.8)/10 (*p* = 0.262) increased significantly only with perturbation training. Scores for sensory orientation, that were high at baseline, did not change significantly with any training (6.0 (1.0) to 6.0 (0)/6 (*p* = 0.059) vs 4.5 (2.0) to 5.4 (1.8)/6 (*p* = 0.336)).

**Table 2:** Within and between group comparisons of outcome measures

		Perturb Group (mean (IQR), *n* = 10)	NonPerturb Group (walking-only training) (mean (IQR), *n* = 8)	NonPerturb Group (perturbation training) (mean (IQR), *n* = 7)
**Dynamic balance (Mini-BESTest) (/28)**	Pre	20.0 (2.8)	16.5 (6.3)	18.5 (4.0)
	Post	23.0 (2.5)	17.0 (3.3)	20.0 (3.5)
Within group comparison pre/post training	Effect Size	.63	.21	.45
	*P* value	**.005**	.932	.089
Between group comparison vs Perturb group post training	*P* value		**.007**	.069
Within NonPerturb group post training	*P* value			**.042**
**Comfortable over ground speed (10 m walk test, in m/s)**	Pre	0.90 (0.31)	0.96 (0.51)	0.83 (0.47)
	Post	1.05 (0.50)	0.93 (0.35)	1.05 (0.44)
Within group comparison pre/post training	Effect Size	.46	.26	.47
	*P* value	**.038**	.292	.075
Between group comparison vs Perturb group post training	*P* value		.594	.807
Within NonPerturb group post training	*P* value			**.018**
**Fast over ground speed (10 m walk test, in m/s))**	Pre	1.36 (0.58)	1.25 (0.47)	1.30 (0.54)
	Post	1.48 (0.75)	1.26 (0.58)	1.33 (0.45)
Within group comparison pre/post training	Effect Size	.60	.13	.47
	*P* value	**.007**	.612	.080
Between group comparison vs Perturb group post training	*P* value		.424	.626
Within NonPerturb group post training	*P* value			.141
**Paretic knee extensors, maximal strength (Dynamometry, in Nm)**	Pre	94.5 (46.6)	113.9 (45.9)	108.0 (26.6)
	Post	139.1 (51.4) (*n* = 7)	123.2 (49.2)	106.3 (40.6)
Within group comparison pre/post training	Effect Size	.59	.07	.32
	*P* value	**.028**	.779	.237
Between group comparison vs Perturb group post training	*P* value		.643	.482
Within NonPerturb group post training	*P* value			.612
**Non-paretic knee extensors, maximal strength (Dynamometry, in Nm)**	Pre	157.7 (64.8)	148.7 (39.2)	150.7 (19.4)
	Post	183.0 (52.0) (*n* = 7)	138.5 (52.3)	162.5 (37.7)
Within group comparison pre-post training	Effect Size	.58	.17	.27
	*P* value	**.028**	.484	.310
Between group comparison vs Perturb group post training	*P* value		.247	.848
Within NonPerturb group post training	*P* value			.735
**Balance confidence (ABC, /100)**	Pre	75.9 (31.3)	65.9 (17.5)	66.6 (19.4)
	Post	76.6 (35.4)	75.2 (22.5)	76.9 (10.3)
Within group comparison pre/−post training	Effect Size	.52	.46	.54
	*P* value	**.021**	.063	**.042**
Between group comparison vs Perturb group post training	*P* value		.657	.922
Within NonPerturb group post training	*P* value			**.043**
**Reintegration to normal living index (RNLI, /22)**	Pre	2.5 (4.0)	2.0 (1.3)	1.0 (1.5)
	Post	2.0 (2.5) (*n* = 9)	1.5 (2.8)	0.0 (1.5)
Within group comparison pre/post training	Effect Size	.46	.00	.46
	*P* value	**.040**	.100	.083
Between group comparison vs Perturb group post training	*P* value		.588	.403
Within NonPerturb group post training	*P* value			.066

*IQR* Interquartile range

Perturbation training resulted in a significant improvement in balance confidence in the Perturb group (from 75.9 to 76.6%) (Table 2). Maximal strength increased at the paretic (+ 47.0%) and non-paretic (+ 16.0%) knee extensors in Perturb group, but not in the NonPerturb group (less than 8.1% increase) (Table 2). RNLI results improved significantly with a mean score reduction in the Perturb group only (Table 2). Post-training comparisons between the Perturb group and the NonPerturb walking-only group showed only a difference in dynamic balance (Mann-Whitney U *p* = .006). Perturbation training in the Perturb group had a large effect size with respect to most variables (ES > .46). However, walking-only training in the NonPerturb group had little (ES < .26) to no effect size for most variables, except on balance confidence (ES = .46) (Table 2, Fig. 4).

### Effects of secondary perturbation training in the NonPerturb group

All but one of the NonPerturb participants (*N* = 7) crossed over to participate in the secondary training with perturbations. There was no difference between post-walking-only training and before perturbation training in NonPerturb group (Wilcoxon *p* ≥ .236). Unpredictable gait perturbation training in the NonPerturb group significantly improved balance confidence (Table 2), with a trend toward a significant difference in dynamic balance, comfortable and faster over-ground speeds (Table 2) and RNLI results (Table 2). Unpredictable gait perturbation training did not improve maximal knee extensor strength on the paretic and non-paretic sides. Medium to large effect sizes were found for all variables (ES > .45) except for maximal paretic and non-paretic knee extensor strength (ES ≤ .31). There was no significant difference between the Perturb and NonPerturb groups after perturbation training (Mann-Whitney U *p* ≥ .069); however, dynamic balance, walking at a comfortable speed, and ABC (Table 2) improved (Wilcoxon *p* ≤ .043) after the perturbation program compared to after the walking-only program in the NonPerturb group. Maximal knee extensor strength never changed in this group (Wilcoxon *p* ≥ .612). Reintegration to Normal Living index results showed a tendency toward a larger improvement after the perturbation training program than after the walking-only training program in NonPerturb group (Wilcoxon *p* = .066) (Fig. 4).

### Effects after six weeks

At the 6-week follow-up, there was no difference in balance confidence (ABC score) or community reintegration (RNLI) compared to post-training for any type of training in either group (Wilcoxon *p* ≥ .223). However, community reintegration did not differ from pre-training values (Wilcoxon *p* < .271), contrary to balance confidence that was still different from pre-training in the Perturb group and after walking-only in the NonPerturb group (Wilcoxon *p* < .047, but *p* = .345 for NonPerturb perturbation training).

### Participant satisfaction

**Fig. 5:**
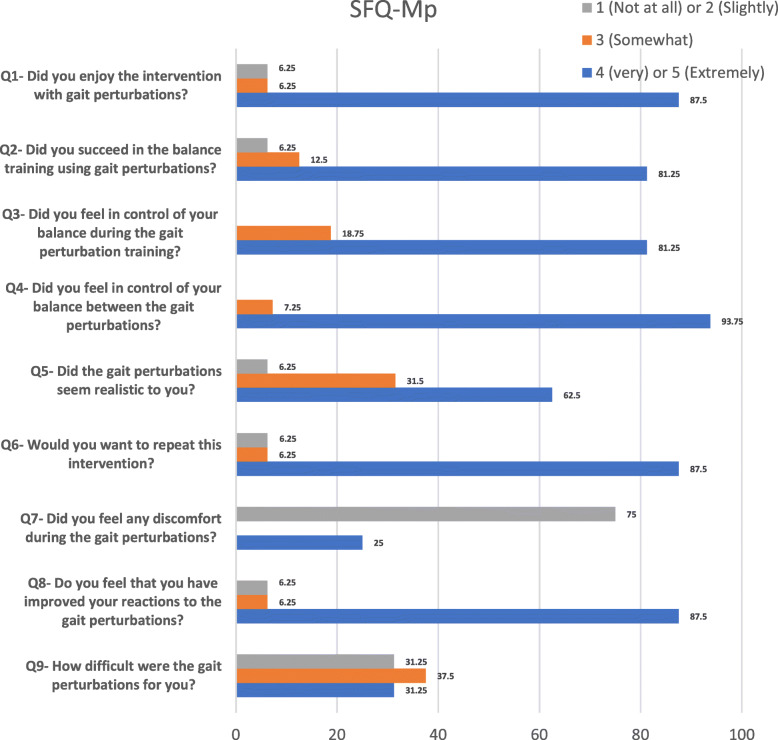
Responses to Short Form Questionnaire-Modified for Perturbations (SFQ-Mp) for participants who attended perturbation training (Perturb: *n* = 10, NonPerturb: *n* = 7), expressed an a percentage (%) of total responses

Participants were generally satisfied with the perturbation training program as more than 62.5% (10/16) of them answered “very” or “extremely” when answering items 1–6 and 8 on the SFQ-Mp questionnaire (Fig. 5). Seventy-five percent of participants selected “not at all” and “slightly” when answering the question about “feeling discomfort.” Thirty-seven point five % (6/16) were neutral about the difficulty level used in the perturbation training program, while the rest of the participants were split between “very” or “extremely difficult” (31.25% (5/16)) and “not at all” or “slightly difficult” (31.25%).

## Discussion

The results of this pilot study support the clinical effectiveness of unpredictable gait perturbations compared to walking-only treadmill training in improving dynamic balance and gait abilities in individuals with chronic stroke. Perturbation training had a significant and large effect on most variables in the Perturb group compared to no effect of walking-only in the NonPerturb group. In addition, large balance and gait improvements were also observed with perturbation training in the control (NonPerturb) group after the walking-only training program failed to produce improvements in balance and gait. The NonPerturb group results also underscore the superiority of the perturbation program, as the lack of improvement during the walking-only training program was not due to the inability of the control participants to improve (i.e. they were not at their maximum in their balance and gait abilities prior to the walking-only program).

To match variables across groups before training, we had to allocate the first nine participants to the Perturb group, which could have potentially increased the selection bias [44]. However, this did not deviate from the randomization method. This occurred because new potential participants did not match the characteristics of the participants previously included in the experimental group, and could thus not be allocated to the control group. Also, two participants allocated to the NonPerturb group could not participate in the control, walking-only, training program after randomization (the one participant had new medical issues, not related with the project and the other was concomitantly diagnosed with cancer). Therefore, despite no pre-training statistical difference between the groups, comfortable speed (.1 m/s) and RNLI (4%) were initially higher in the NonPerturb group, and dynamic balance was better (1.7/28) in the Perturb group. This likely affected between-group comparisons, mostly in favor of the walking-only training in the NonPerturb group. As a result, positive results presented here had to be strong to reach statistical significance.

Improvement in dynamic balance was the sole statistically significant difference, with a large effect size, between perturbation training in the Perturb group and walking-only training in the control, NonPerturb group. In comparison, walking-only training did not improve dynamic balance at group or individual levels. More specifically, perturbation training had a specific effect on reactive postural control and dynamic gait sub-systems, contrary to walking-only. Since reduced balance abilities is a major risk factor for falls, [10] these results may have an impact on the risk of falls; however, the number of falls post-training was not evaluated in the present study. Previous perturbation training programs among individuals with chronic and subacute stroke, consisted of 10–12 sessions during which perturbations were applied manually or by the antero-posterior or mediolateral translation of the support platform or treadmill in a standing position or during gait [20–22, 45, 46]. Improvements in reactive and proactive balance control after perturbation training [21, 45, 46], also observed in our results, and specifically for reactive activity and dynamic gait, likely explains better balance abilities, as determined by clinical evaluations such as the Berg Balance Scale (BBS) or the Timed Up and Go (TUG) test [22]. BBS was not used in this study because we targeted dynamic balance with the perturbation training and because of the ceiling effect of the BBS in individuals post-stroke [3].

In addition to dynamic balance, gait speed also improved in the Perturb group. This improvement could be attributed to perturbation training since the speed on the treadmill was not increased in any group during training. Improvements after perturbation training post-crossover, compared to the absence of improvement post walking-only training in NonPerturb group, emphasize the beneficial effect of perturbation training on abilities in individuals with chronic stroke. Punt et al. also reported comfortable speed improvements, similar to our study (+ 0.16 m/s) [20]. However, their training program included periods of gait at higher than comfortable gait speed, [19–22] which could have equally led to improved gait speed as much as did perturbations. It is therefore possible that the challenge posed by gait perturbations in the present study is a strong enough stimulus to improve the abilities required for both balance and gait. Contrary to our hypothesis, walking-only training did not improve gait abilities in the control group. This is likely explained by the good walking abilities of the participants pre-training (mean speed of 1.0 m/s) and confirms the limited challenge that walking at a comfortable speed posed for this group.

Secondary outcomes also noticeably improved. Balance confidence increased with both types of training. Other studies found similar results between + 3.6 points and 10 points on the ABC scale, over 10 to 30 training sessions using moveable platform perturbations, [46] manual perturbation in a standing position, [19] and an agility exercise program designed to challenge dynamic balance [14]. However, this improvement may not be directly related to perturbation as balance confidence increased by similar amounts between perturbation- and walking-only training. The combination/duration of the two trainings periods that the NonPerturb group received may also have potentialized the effect on balance confidence. Longer periods of intervention indeed tend to provide greater improvement in balance confidence in older adults [47]. Lastly, maximal knee extensor strength improved in the Perturb group, but not during the secondary perturbation training in the NonPerturb group. However, dynamic balance improved in both groups after perturbation training. Higher maximal strength may thus not be a prerequisite for balance improvement, as underscored by the conflicting evidence of the effect of strength training on balance in a previous meta-analysis [48].

Perturbation training in both groups led to an increased level of community reintegration as evaluated by the RNLI, with some sustained effect at 6 weeks. Improvement in RNLI scores after perturbation training supports the fact that improvements in balance abilities translated into better participation and mobility in the community. Previous studies that used progressive standing perturbations [19] or low-intensity gait perturbations [20] did not show transfer to daily-life mobility. It is possible that the more intense and higher number of gait perturbations used in our study may have had a better effect on mobility and thus on community reintegration. These effects might be explained by the fact that these perturbations were applied during gait, at various and sometimes high intensities, in an unpredictable manner, and required adapted stepping reactions that could be used during loss of balance in daily life [49]. Such an effect in daily life may also explain the sustained effect at 6 weeks. It is to be noted that this result may also have been affected by the large variability of the pre-training mean score in the Perturb group. Also, both groups had an already good level of reintegration (i.e. low score) observed pre-training. Further studies are necessary to confirm increased community integration through objective measures and longer-term follow-up.

Despite the loss of balance induced by the perturbations and the intense postural reactions they triggered, a large majority of the participants felt in control during and between the perturbations and enjoyed the perturbation program, with very little discomfort. This might have been facilitated by the design of the program, with repeated perturbations followed by unpredictable perturbations, and by the possibility of producing small intensity perturbations at first, which then increased according to the participant’s comfort level. Such progression in the intensity of the perturbations was facilitated by the use of a treadmill. However, progression in level of difficulty might need to be more personalized as the perception of difficulty was reported by our participants as being between “Not difficult” and “Extremely difficult.” Despite this wide range of difficulty perception, most participants thought they were successful and improved their balance abilities during the training. The only other subjective evaluation found in the literature concerned the difficulty of the perturbation, which was rated as high as 7/10, with 10 representing a very difficult challenge [21]. In that study, only medio-lateral perturbations were used during gait, at the highest intensity possible without inducing a fall.

## Limitations

Participants were allocated to groups by covariate adaptive randomization due to small sized groups, resulting in no statistical differences pre-training. However, minor pre-training differences in clinical scores may have limited the demonstration of superiority of the perturbation training over walking-only training across all primary outcome measures, rather than just for balance abilities, despite the absence of effect of the walking-only training for these outcomes.

Secondly, fall-related data was not collected after the study. However, though most of the participants were not prone to falling, their balance abilities were below normal, which is one of the main risk factors for falls [10]. In addition, because of their reduced balance confidence, the participants may have reduced their dynamic activities to reduce fall risks, thus affecting the pre-training risk of falls. Since being prone to falling was not an inclusion criterion, evaluating the number of falls pre- and post-training was not considered useful, particularly given the short follow-up period. To become a promising method for reducing falls, as already observed in other populations other than stroke, further large sample size studies are necessary to complete previous inconclusive findings [20, 50]. For example, different intensities of perturbation and longer follow-up periods should be tested in individuals post-stroke with various levels of deficits. Note that one of our participants initially presented with low gait speed (0.39 m/s) and balance (11/28 on the MiniBESTest) abilities. This participant attended all the training sessions with perturbations, improved his balance abilities (20/28 on the MiniBESTest) and enjoyed the perturbation training for its level of challenge. This highlights the feasibility of using perturbations that can be easily adapted to participants’ abilities.

Since both repeated and unpredictable perturbations were applied in each training session, it was not possible to determine which kind or combination of perturbations was more effective in improving balance and gait abilities. It is also possible that the number of perturbations was higher than necessary for maximizing balance abilities. However, unpredictable balance perturbations are closer to real-life conditions and are thus conceptually warranted.

Finally, split-belt treadmills are designed to cause unpredictable perturbations for clinical and rehabilitation purposes using a complex control system, [51] but their availability in clinical settings is rare, which may hamper the generalization of this approach. The large number of perturbations produced also makes this intervention difficult to apply in clinical practice due to the length of the training session.

## Conclusion

Perturbation gait training improved both physical and psychological aspects of balance in individuals with chronic stroke. The results emphasize the specific effect intense and unpredictable perturbations have over the effect of gait-only training on a treadmill. Large effect sizes obtained in the present study support the clinical effectiveness of this task-specific program in individuals with chronic stroke. Evaluation of this program, including variation in the type and number of perturbations generated, with a larger sample size, long-term follow-up and fall monitoring, is now warranted.

## Data Availability

The datasets used and/or analysed during the current study are available from the corresponding author on reasonable request.

## Abbreviations

ABC: Activity-specific Balance Confidence
BBS: Berg Balance Scale
CIUSSS: Centre Intégré Universitaire de Santé et de Services Sociaux
CMSA: Chedoke McMaster Stroke Assessment
CONSORT: Consolidated Standards of Reporting Trials
ES: Effect size
Fig.: Figure
MCID: Minimal Clinically Important Difference
Mini-BESTest: Mini-Balance Evaluation Systems Test
N: Number
NParetic: Non-paretic
Non-Perturb: Name of the control group where participants received the gait training program without perturbation in the first part, and were then offered to participate to the training program with gait perturbation once they finished gait training program without perturbations.
Perturb: Name of the experimental group where participants received the gait perturbation training program.
RNLI: Reintegration to Normal Living Index
SFQ-Mp: Short Feedback Questionnaire, modified for perturbations
TIDieR: Template for Intervention Description and Replication
TUG: Timed Up and Go
